# The Book World of Medicine and Science

**Published:** 1904-08-20

**Authors:** 


### The Book World of Medicine and Science.

Unconscious Therapeutics; or The Personality of the Physician. By Alfred T. Schofield, M.D. (London: J. and A. Churchill. Pp. 317. Price 5s. net.)

Dr. Schofield appears to have been in difficulty about the title of this volume, for he has sent it forth with two designations. Of these we prefer the second, for it better defines the contents of the book. Dr. Schofield puts forward the proposition that the personality of the physician is an important therapeutic agent, and that, therefore, the special characteristics which are likely to be potent in this respect should be systematically cultivated by individual practitioners and taught in the schools. By such a plan, he believes, a great deal would be done to diminish the popularity of all forms of quackery. The public now resorts to charlatans and quack nostrums of all kinds because they have profound faith in these agents, and their confidence alone is sufficient to materially aid recovery from their ailments, whether these be real or imaginary. If, thinks Dr. Schofield, medical men were but able to exercise the same kind of mental persuasion in addition to the other means of treatment at their disposal they would no longer have to suffer the humiliation of seeing the charlatan prosper at their expense. There is much that is debatable in the book which, however, we can recommend to everyone who takes an interest in the wider issues of the profession of medicine.

Alcohol: Its Place and Power in Legislation. By Robinson Souttar, M.A., D.C.L. (London: Hodder and Stoughton. Pp. 259. Price 3s. 6d.).

This book embodies a dissertation presented by Dr. Souttar when qualifying for the degree of doctor of civil law at Oxford University; and it forms a useful compendium of the laws that have been made in England and certain other countries affecting the production and consumption of alcoholic beverages, together with valuable criticisms, chiefly from a legal standpoint, of the various remedial measures proposed or adopted at different times and in different places to limit the consumption of alcohol. Especially interesting are the chapters dealing with the right of the State to interfere with the liquor traffic, the law of licensing in England, Scotland, the United States, Canada, Scandinavia and Russia, compensation to publicans on withdrawal of licenses, public house trusts, and the habitual inebriate. It is noteworthy that Dr. Souttar brings forward some weighty arguments against the operations of the public house trusts which have come into such prominence lately, and his remarks on this subject, as, indeed, the whole volume, should be carefully studied by all who are concerned with the many problems of the drink question.

Practical Prescribing and Dispensing for Medical Students. By William Kirby. (Manchester: At the University Press. 1904. Pp. 169. Price 4s. 6d. net.)

This is a very sensible and useful manual. It recognises the fact that the training of the medical student in pharmacy has a purpose different from that aimed at in the education of the chemist and druggist. Thus it provides a series of practical exercises designed to make the student familiar with the physical and chemical properties of the more commonly used remedies, and to render him competent to combine drugs in the form of prescriptions. We have nothing but praise for the manner in which this scheme is carried out, and were such a discipline compulsory on all medical students we should hear less than we are accustomed to in the present day of a decline in the art of prescribing. This would certainly mean a lessened activity on the part of the manufacturer of new remedies and ready-made prescriptions, but we cannot pretend to have any tears to spare for his misfortunes.

Biographic Clinics. Vol. II. By George M. Gould, M.D. (London: Rebman, Limited. 1904. Pp. 392.)

We cannot say that Dr. Gould is any more convincing in the present volume than he was in the former one with his theory of eye-strain as the one cause of the sufferings of genius. Doubtless headaches frequently depend upon ocular defects, and great men have suffered from headaches, but there is certainly no proper evidence for believing, as Dr. Gould would have us, that George Eliot, George Henry Lewes, Wagner, Parkman, Jane Welch Carlyle, Spencer, Whittier, Margaret Fuller Ossoli, and Nietzsche were all victims of uncorrected errors of refraction. It might be urged with equal force that their neuralgias and dyspepsias would have been amenable to skilful dentistry or to careful dieting.

The Pocket-Book of Temperature Charts. London: (The Scientific Press, Limited. Price 6d. net.)

The want of a set of temperature charts such as this pocket-book provides must have frequently been keenly felt by all medical men and students who make a habit of keeping notes of their cases. Each book contains seventeen twelve-hour and eight four-hour charts. The pages have perforated attachments, so that when filled up the charts can be torn off and placed in the case-book. These charts might very well be used, we think, in the hospitals, for they would materially help to reduce the size of the usually unwieldy records.

Radium and All About It. By S. R. Bottone. (London: Whittaker and Co., 1904. Pp. 96. Price 1s. net.)

This is a handy little book, consisting of four chapters, in which are discussed the history of the discovery of radium, the mode of its detection and extraction, its properties, and some theoretical considerations to which its discovery gives rise. The paper and printing are good, and the booklet is written in a lucid style. We can recommend it to everyone who is not yet acquainted with the properties of radium and its theoretical importance in the world's economy.

Easily Grown Hardy Perennials. By George H. Vos, B.A., etc. (London: W. H. and L. Collingridge, 1904. Pp. 476. 255 illustrations. Price 5s.)

The pleasures of a garden no doubt depend largely on the amount of personal labour bestowed upon it by the owner, and to many professional men there is no more delightful recreation after a hard day of anxiety and toil than a turn of manual labour in a garden. To such we can strongly recommend this book. It not only gives very good hints on the best hardy perennials to select and how to grow them, but it is also a guide on how to keep a pleasant flower garden without such an amount of labour as annuals and delicate exotic plants would demand. The numerous illustrations, reproduced from photographs, are almost beyond praise.

